# Dairy DigiD: a keypoint-based deep learning system for classifying dairy cattle by physiological and reproductive status

**DOI:** 10.3389/frai.2025.1545247

**Published:** 2025-08-22

**Authors:** Shubhangi Mahato, Hanqing Bi, Suresh Neethirajan

**Affiliations:** ^1^Faculty of Computer Science, Dalhousie University, Halifax, NS, Canada; ^2^Faculty of Mathematics, Dalhousie University, Waterloo, ON, Canada; ^3^Faculty of Agriculture, Agricultural Campus, Dalhousie University, Truro, NS, Canada

**Keywords:** dairy welfare, cattle classification, digital livestock farming, dairy cow biometrics, precision dairy farming

## Abstract

Precision livestock farming increasingly relies on non-invasive, high-fidelity systems capable of monitoring cattle with minimal disruption to behavior or welfare. Conventional identification methods, such as ear tags and wearable sensors, often compromise animal comfort and produce inconsistent data under real-world farm conditions. This study introduces Dairy DigiD, a deep learning-based biometric classification framework that categorizes dairy cattle into four physiologically defineda groups—young, mature milking, pregnant, and dry cows—using high-resolution facial images. The system combines two complementary approaches: a DenseNet121 model for full-image classification, offering global visual context, and Detectron2 for fine-grained facial analysis. Dairy DigiD leverages Detectron2’s multi-task architecture, using instance segmentation and keypoint detection across 30 anatomical landmarks (eyes, ears, muzzle) to refine facial localization and improve classification robustness. While DenseNet121 delivered strong baseline performance, its sensitivity to background noise limited generalizability. In contrast, Detectron2 demonstrated superior adaptability in uncontrolled farm environments, achieving classification accuracies between 93 and 98%. Its keypoint-driven strategy enabled robust feature localization and resilience to occlusions, lighting variations, and heterogeneous backgrounds. Cross-validation and perturbation-based explainability confirmed that biologically salient features guided classification, enhancing model transparency. By integrating animal-centric design with scalable AI, Dairy DigiD represents a significant advancement in automated livestock monitoring-offering an ethical, accurate, and practical alternative to traditional identification methods. The approach sets a precedent for responsible, data-driven decision-making in precision dairy management.

## 1 Introduction

Digital livestock farming has emerged as a transformative strategy in addressing the global demand for sustainable, efficient, and ethically grounded agricultural practices (Neethirajan, 2021). This transformation has been strongly fueled by the integration of deep learning techniques across various aspects of precision cattle farming, highlighting applications ranging from health monitoring to behavioral analysis (Mahmud et al., 2021). A trend further reinforced a significant increase in PLF publications worldwide, highlighting the field’s rapid evolution toward data-driven and automated livestock management solutions (Jiang et al., 2023). At the heart of this transformation lies the ability to monitor and categorize individual animals with precision, enabling targeted health interventions, optimized resource use, and improved animal welfare outcomes (Schillings et al., 2021). Recent work has also demonstrated the application of high-precision spatial tracking systems for monitoring Holstein cattle movements, illustrating the expanding role of AI in holistic herd (Luo et al., 2024). Accurate classification of livestock into relevant physiological categories is fundamental to the development of scalable precision management systems that support individualized care, behavioral monitoring, and informed decision-making (Neethirajan, 2024a, 2024b).

Historically, cattle identification and categorization have relied heavily on physical markers such as ear tags and branding, standardized through regulatory agencies like the Canadian Food Inspection Agency (CFIA). While these methods are entrenched in industry practice, they present several drawbacks: they are invasive, potentially induce stress, and may disrupt natural behaviors (Schnaider et al., 2022). Even more modern tools like RFID collars and accelerometers can interfere with routine activities, posing concerns about data integrity and animal welfare (Neethirajan, 2024a, 2024b).

To overcome these limitations, recent efforts have explored biometrically driven, non-invasive alternatives such as muzzle prints and coat pattern analysis (Hossain et al., 2022; Li et al., 2022; Chen et al., 2021; Manoj et al., 2021; Gerdan Koc et al., 2024), with critical reviews supporting their potential in cattle identification (Kaur et al., 2022) and recent advances demonstrating the efficacy of AI-based muzzle recognition systems for reliable cattle identification (Islam et al., 2024). Similarly, Kusakunniran et al. (2023) proposed a real-time cattle biometric identification system (Cattle AutoID) that leverages facial patterns for individual recognition, highlighting the increasing deployment of AI-driven solutions in livestock settings. Historical approaches like nose print-based identification also laid foundational work for facial biometrics in livestock (Bello et al., 2020). Early computer vision approaches employing techniques like Scale-Invariant Feature Transform (SIFT) with Random Sample Consensus (RANSAC) leveraged unique facial patterns in cattle for individual identification (Awad et al., 2013). These early efforts laid the groundwork for what is now considered a new frontier in animal biometrics, where facial features serve as non-invasive, reliable markers for individual cattle recognition and classification (Kumar and Singh, 2020). Other handcrafted feature techniques, such as Local Binary Patterns (LBP), were also explored for cattle face recognition, demonstrating potential under constrained conditions (Cai and Li, 2013). However, these methods struggled under real-world conditions characterized by inconsistent lighting, occlusion, background clutter, and pose variability (Scott et al., 2024; Araújo et al., 2025; Li et al., 2021), limiting their scalability and robustness in commercial dairy environments.

The emergence of classical machine learning methods such as Support Vector Machines (SVM), k-Nearest Neighbors (k-NN), and Artificial Neural Networks (ANN) enabled the automated classification of biometric features, thereby reducing manual annotation efforts (Hossain et al., 2022). Nevertheless, these models depended heavily on handcrafted features, limiting their generalization capacity across varied farm conditions. Their performance was further constrained by small datasets and rigid preprocessing pipelines, as noted by Cockburn (2020).

The advent of deep learning, particularly Convolutional Neural Networks (CNNs), has significantly advanced visual feature extraction and classification in livestock management. Architectures like ResNet (He et al., 2016), YOLO (Redmon et al., 2016; Redmon and Farhadi, 2018; Bochkovskiy et al., 2020; Yılmaz et al., 2021), and VGG16 (Simonyan and Zisserman, 2014) have demonstrated high performance in image-based cattle recognition tasks. For example, El-Henawy et al. (2016) and Andrew et al. (2017) reported notable improvements using CNNs enhanced with texture and region-based analysis for Friesian cattle, while de Lima Weber et al. (2020) achieved classification accuracies approaching 99% in controlled settings. Earlier studies by Yao et al. (2019) explored cow face detection and recognition through automatic feature extraction algorithms, marking foundational steps toward non-invasive biometric identification in livestock.

Weng et al. (2022) further advanced this domain by developing a two-branch CNN architecture tailored for cattle face recognition, demonstrating the field’s progression toward more specialized network designs. Yang et al. (2024) fused RetinaFace with an improved FaceNet to identify individual cows in unconstrained farm environments, highlighting the move toward integrated detection and embedding systems specifically designed for livestock facial recognition. However, despite these advancements, CNN-based systems have their limitations. VGG16, for instance, is computationally intensive and slow to train (Simonyan and Zisserman, 2014), while YOLO, although efficient, may produce false positives for smaller regions such as cattle faces without extensive parameter tuning (Neethirajan, 2024a, 2024b).

Facial biometrics, particularly the detection of consistent landmarks such as eyes, nostrils, ears, and muzzle contours, offers a stable and anatomically grounded alternative to full-body analysis (Qiao et al., 2019; Bergman et al., 2024). These facial keypoints—discrete, localizable points on the animal’s face—allow for the construction of a geometrically meaningful representation that remains relatively invariant across time, environmental conditions, and physiological changes (Rashid et al., 2017). Unlike body posture, which can vary significantly due to gait, pregnancy, injury, or coat shedding, facial features provide reliable identifiers that are well-suited for longitudinal monitoring (Meng et al., 2025). Moreover, facial recognition systems can be seamlessly integrated into existing farm infrastructure (e.g., feeding stations, milking parlors), minimizing additional hardware needs and animal disturbance (Bergman et al., 2024). Recent study highlight the predictive power of facial biometrics in dairy cows, noting that changes in features such as eye openness, muzzle texture, and ear orientation can serve as early indicators of health and stress, underscoring the potential value of integrating facial feature tracking into physiological group classification systems (Mahato and Neethirajan, 2024). Further advancing this field, Meng et al. (2023) demonstrated enhanced cattle face recognition systems capable of distinguishing even unfamiliar individuals, illustrating the growing sophistication of AI-driven biometric approaches in smart livestock management. Other study has shown that non-invasive computer vision approaches can effectively predict both age and welfare status in dairy cows, underscoring their potential as automated veterinary support systems (Fuentes et al., 2022).

Building on these insights, this study introduces Dairy DigiD, a novel deep learning-based biometric classification system designed to categorize dairy cattle into four physiologically meaningful groups: young, mature milking, pregnant, and old. Importantly, this study does not aim to individually identify cattle in a biometric sense (i.e., assigning unique IDs), but instead leverages biometric facial features to perform robust group classification. To explore the most effective method for this task, we performed two distinct deep learning strategies: first, we employed DenseNet121 (Huang et al., 2017) as a baseline model, to evaluate classification performance using full-image inputs; later utilized Detectron2, an advanced object detection and keypoint estimation framework developed by Facebook AI Research (Wu et al., 2019), to focus exclusively on facial regions annotated with 30 anatomical landmarks.

Detectron2’s modular design and precise keypoint detection capabilities enable it to outperform generic CNNs in environments marked by occlusion, shadow, and background variability - conditions common in commercial farms. Unlike methods that rely on rectangular bounding boxes, Detectron2 uses pixel-level segmentation and keypoint mapping to extract fine-grained, anatomically relevant features. This allows for richer, more explainable model behavior and more consistent predictions under challenging visual conditions (Wu et al., 2019).

In this study, we address two primary research objectives:

1. To investigate whether advanced deep learning methods - starting with a full-image classification approach (DenseNet121) and later to a keypoint-driven facial biometrics approach (Detectron2) - can effectively categorize dairy cattle under realistic farm conditions.
2. To compare the performance and interpretability of these two approaches, emphasizing the robustness and contextual specificity afforded by keypoint-based classification.

**Figure 1:**
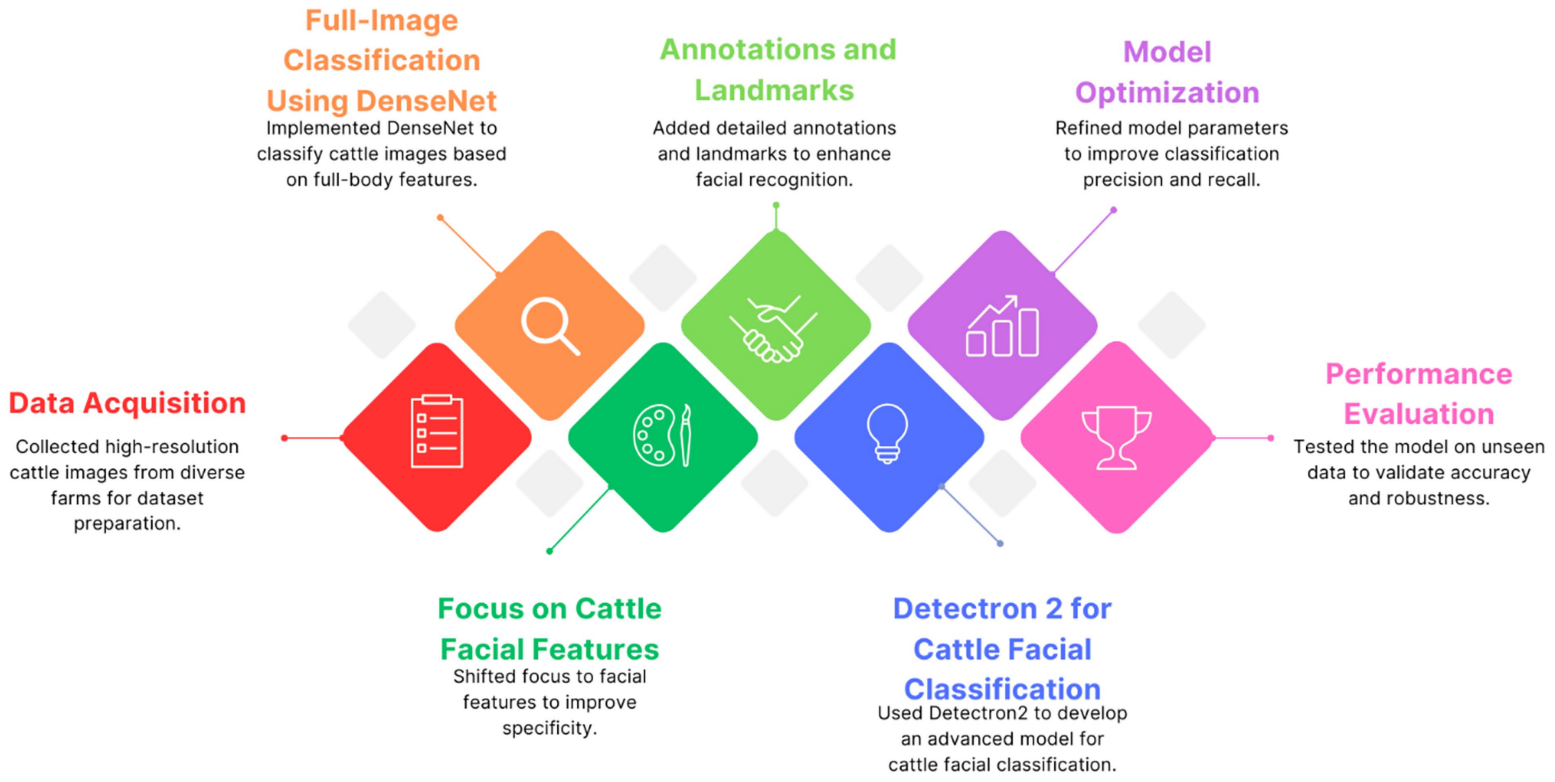
Workflow diagram illustrating the key steps in developing the Dairy DigiD cattle image classification model, from data collection to model evaluation.

While our original scope included comparisons with other architectures such as VGG16 and YOLO, this study focuses on DenseNet121 as a representative baseline for CNN-based global image classification. This refinement enables a more targeted and in-depth comparison with Detectron2, preserving methodological clarity. A broader benchmarking analysis remains an important avenue for future work. The conceptual overview and methodological pipeline of our system are presented in Figure 1.

## 2 Dataset and methodology

Our study draws on a carefully curated dataset designed to reflect the diversity, environmental complexity, and physiological variability of dairy cattle populations across Nova Scotia and New Brunswick, Canada. The dataset comprises over 8,700 high-resolution images, representing approximately 600 Holstein and Jersey cows, and supports the development of scalable AI models for precise, non-invasive cattle classification.

### 2.1 Ethical approvals

All procedures were reviewed and approved by the Dalhousie University Ethics Committee (Protocol 2024–026). Data collection involved no physical contact with animals. Participating farm owners were fully informed of the study’s objectives and provided written consent. In adherence to ethical guidelines, data were collected exclusively through passive image and video capture.

### 2.2 Cattle demographics and dataset composition

Images were collected from commercial farms housing Holstein and Jersey cattle, with herd sizes ranging from 60 to 110 animals. Animals were photographed in natural farm environments including barns, pastures, and open yards. This contextually rich data was critical for training models to perform reliably under diverse real-world conditions. Images were categorized based on physiological state:

- Young cows (<2 years): 520 images
- Mature milking cows: 5,740 images
- Pregnant cows: 1,290 images
- Dry cows: 1,197 images

### 2.3 Data collection methods

Visual data were collected using consumer-grade high-resolution cameras (Samsung S21, iPhone 13/14), capturing multiple poses and lighting conditions. Over 6,000 images captured side, frontal, and angled views. Selected video frames were extracted to supplement image diversity and improve environmental robustness.

### 2.4 Data partitioning

To ensure robust model training and evaluation, the dataset was partitioned into training (70%), validation (10%), and independent test (20%) sets. The validation set was used during training for hyperparameter tuning and early stopping, while the independent test set was reserved exclusively for final performance assessment on unseen data. Stratified sampling was employed during partitioning to maintain approximate class balance across all subsets. However, due to the inherent class imbalance in the original dataset - particularly the underrepresentation of the “Pregnant Cow” category - perfectly equal distribution was not feasible. To address this, targeted data augmentation was applied only to the training set to enhance model learning without introducing data leakage into the validation or test sets. This approach ensured that both the validation and test sets remained representative of real-world distributions, preserving their value as unbiased evaluation tools. For Detectron2-specific evaluation, a 5-fold cross-validation protocol was applied to a curated subset of the training data. Each fold preserved relative class distributions via stratified sampling, allowing for consistent benchmarking while accounting for variability in image quality and environmental conditions (see Section 4.5).

### 2.5 Labeling and annotation

To create a robust dataset for biometric classification, three animal-science experts annotated approximately 8,700 cattle images using the Computer Vision Annotation Tool (CVAT). Each image was labeled with 30 anatomically derived facial landmarks, systematically distributed across the eyes (points 1–8), ears (9–18), muzzle (19–24), and head contours (25–30). This landmark schema, based on established protocols (Han et al., 2022; Shojaeipour et al., 2021), enables physiologically relevant feature extraction under varying farm conditions—supporting pose normalization, individualized biometric marker identification, and geometric alignment for downstream machine learning models.

Prior to the main annotation phase, a 300-image calibration round was conducted in which all annotators independently placed keypoints, allowing us to directly assess the consistency and spatial precision of landmark placement. Inter-annotator agreement was quantified using Fleiss’ *κ*, producing a strong value of 0.87, which reflects high reliability in keypoint localization across experts. Discrepancies in this round were carefully reviewed and codified into standardized anatomical guidelines. Annotations throughout the project were performed manually—CVAT provides organizational tools but does not automate keypoint placement—ensuring that all labels reflected expert anatomical judgment.

After calibration, one thoroughly trained annotator completed the full dataset, with rolling quality control: a second expert spot-checked every 10th batch (approximately 5% of the data), making corrections when minor discrepancies (<2% of keypoints) were detected. Additionally, images associated with high training loss during initial model development were manually re-inspected to identify rare labeling errors.

Simultaneously, each image was associated with a physiological category label corresponding to four distinct groups: young, mature milking, pregnant, and dry cows. Importantly, these class assignments were not determined by the annotators from image review, but were instead sourced from up-to-date veterinary records and on-farm information provided by the farmers and animal caretakers at the time of image capture. This approach ensures that the physiological status of each animal—reflecting age and reproductive stage—was definitively known and objectively recorded, providing a gold-standard ground truth for supervised model training.

Our annotation pipeline balanced thoroughness, biological validity, and efficiency: dual annotation of the full corpus was precluded by resource constraints, but high calibration agreement, transparently manual anatomical labeling, rolling spot-check audits, and the use of farm-verified physiological categories provided rigorous safeguards against label noise.

### 2.6 Computational resources and code availability

All experiments were conducted on Google Colab using NVIDIA Tesla T4 GPUs (16 GB). The environment utilized PyTorch 1.10, CUDA 11.2, OpenCV, and CVAT. Detectron2 was installed from the official Facebook AI Research GitHub repository (Wu et al., 2019).

### 2.7 Data augmentation techniques

To mitigate class imbalance, particularly in the pregnant cow category, we applied extensive augmentation. The original 89 images were expanded to 1,200 via controlled geometric (rotation, flipping, scaling) and color-space transformations. These techniques enhanced model generalization and improved classification performance for underrepresented groups, as reflected by an increase in training F1-score from 0.93 to 0.96.

## 3 Model development and experimental procedures

### 3.1 Overview of the classification pipeline

This study developed a two-stage deep learning pipeline for non-invasive classification of dairy cattle into physiologically meaningful groups based on facial images. In the first stage, DenseNet121 - a convolutional neural network optimized for efficient feature propagation - was trained using facial images, each explicitly assigned to one of four physiological class categories: young, mature milking, pregnant, or dry cow. These category labels were determined objectively from up-to-date veterinary records and farm-provided status information.

DenseNet121 thus learned to discriminate among these general groups based solely on class labels linked to each image, leveraging global visual cues across the animal’s head. In the second stage, a keypoint-based approach was applied using Detectron2, which extracted and utilized 30 anatomical facial landmarks to enhance classification specificity. This approach enabled the model to focus on fine-grained biometric features and reduce sensitivity to background variation.

By comparing the global, label-supervised classification of DenseNet121 with the anatomically focused analysis provided by Detectron2, our sequential pipeline enables rigorous assessment of both holistic and localized visual features under realistic farm conditions.

### 3.2 DenseNet121 full-image classification

#### 3.2.1 Configuration and training

**Figure 2:**
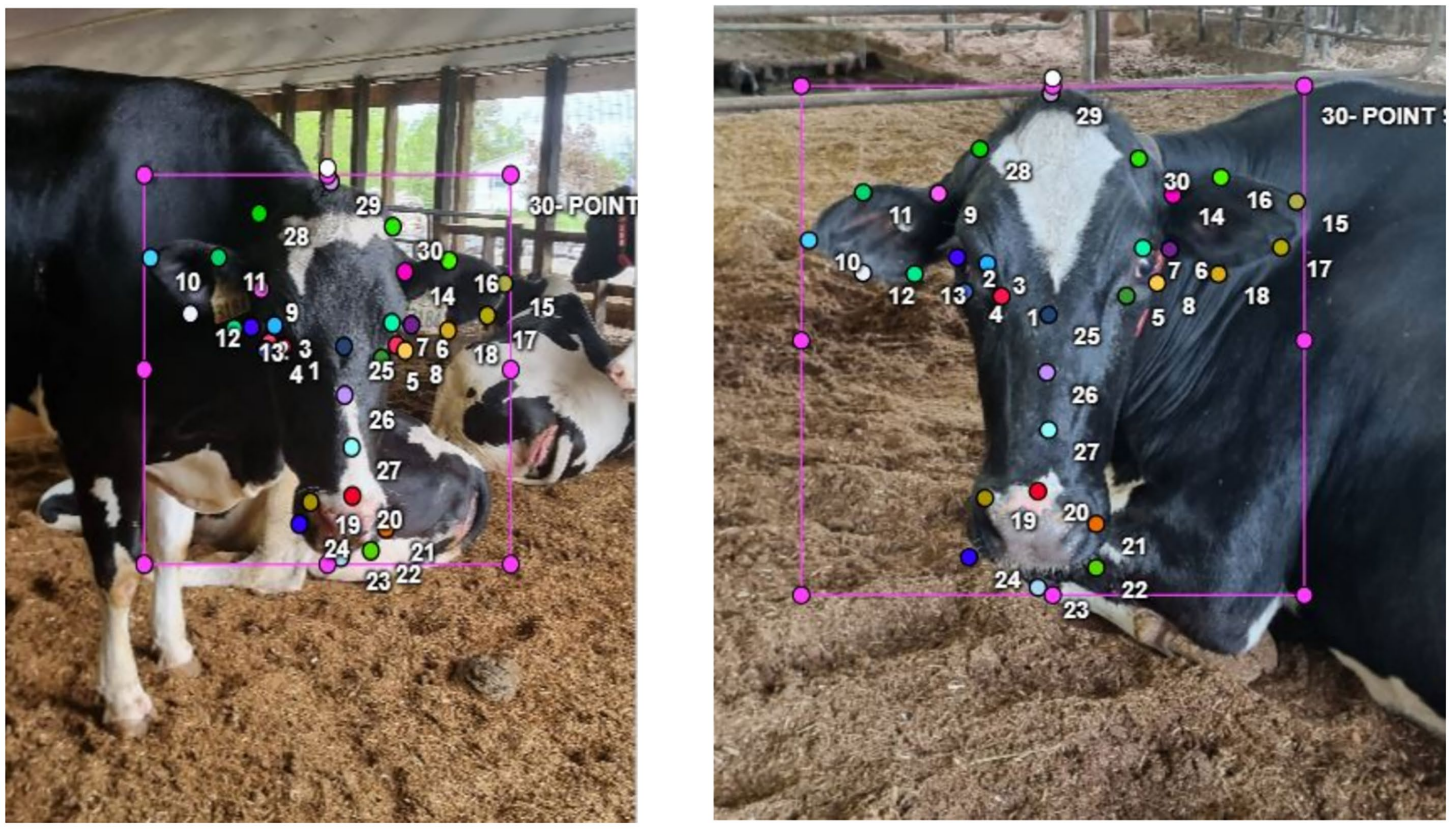
Diagrammatic representation of the 30 annotated facial landmarks on cattle images. Color-coded markers distinguish key regions: eyes, ears, muzzle, and head contour. These landmarks form the basis of the keypoint detection system used in Detectron2.

DenseNet121 was fine-tuned using pre-trained ImageNet weights. Input images were resized to 224 × 224 pixels to match model input dimensions. We used the Adam optimizer with an adaptive learning rate starting at 0.0001, a batch size of 16, and categorical cross-entropy as the loss function. Training proceeded for up to 50 epochs with early stopping based on validation loss to prevent overfitting. The model architecture features dense connections between convolutional layers, which facilitate gradient propagation and enhance learning efficiency (Huang et al., 2017). To examine the internal logic of DenseNet121’s predictions, we applied Gradient-weighted Class Activation Mapping (Grad-CAM) (Selvaraju et al., 2020) (Figure 2).

### 3.3 Facial landmark design and annotation

#### 3.3.1 Detectron2 keypoint integration and training protocol

The 30 anatomically annotated facial landmarks were harnessed to train Detectron2’s Keypoint R-CNN module (Girshick, 2015), enabling the network to learn spatially precise keypoint localization in conjunction with class labels and bounding boxes. This integration allowed the model to focus on high-signal facial features—such as the eyes, ears, muzzle, and head contours—instead of background noise, thereby enhancing classification robustness.

Training was performed over 2,000 iterations with a batch size of 4, using a learning rate of 0.0005 scheduled to decay at 1,500 and 3,000 iterations. The AdamW optimizer (Loshchilov and Hutter, 2017) facilitated adaptive gradient control and improved regularization. Hyperparameters—including learning rate, batch size, and the number of epochs—were empirically tuned to optimize convergence while minimizing overfitting and GPU memory usage. The model was trained for up to 10 epochs, with close monitoring of both training and validation loss curves; convergence was typically reached by epochs 8–9. To further guard against overfitting, an early stopping protocol was implemented with a patience of 2 epochs, ensuring training ended automatically if validation loss plateaued. This strategy guaranteed that the final model reflected the optimal balance between generalization performance and learning depth.

### 3.4 Detectron2 model configuration and architecture

#### 3.4.1 Architectural components

**Figure 3:**
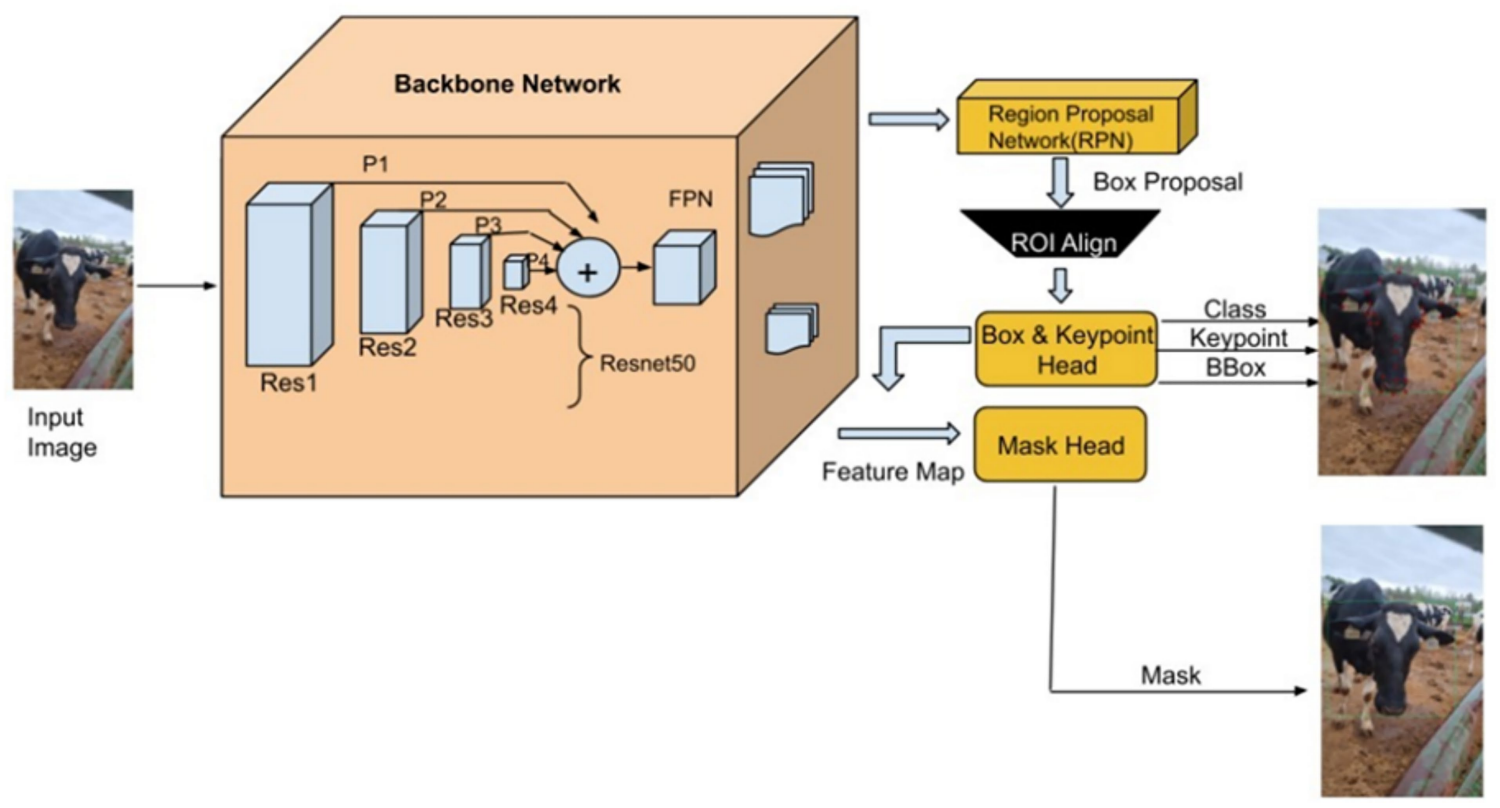
The framework diagram illustrates Detectron2’s integrated architecture for cattle classification. While keypoint detection is emphasized, instance segmentation was employed to refine facial region proposals and remove background interference during pre-processing.

**Figure 4:**
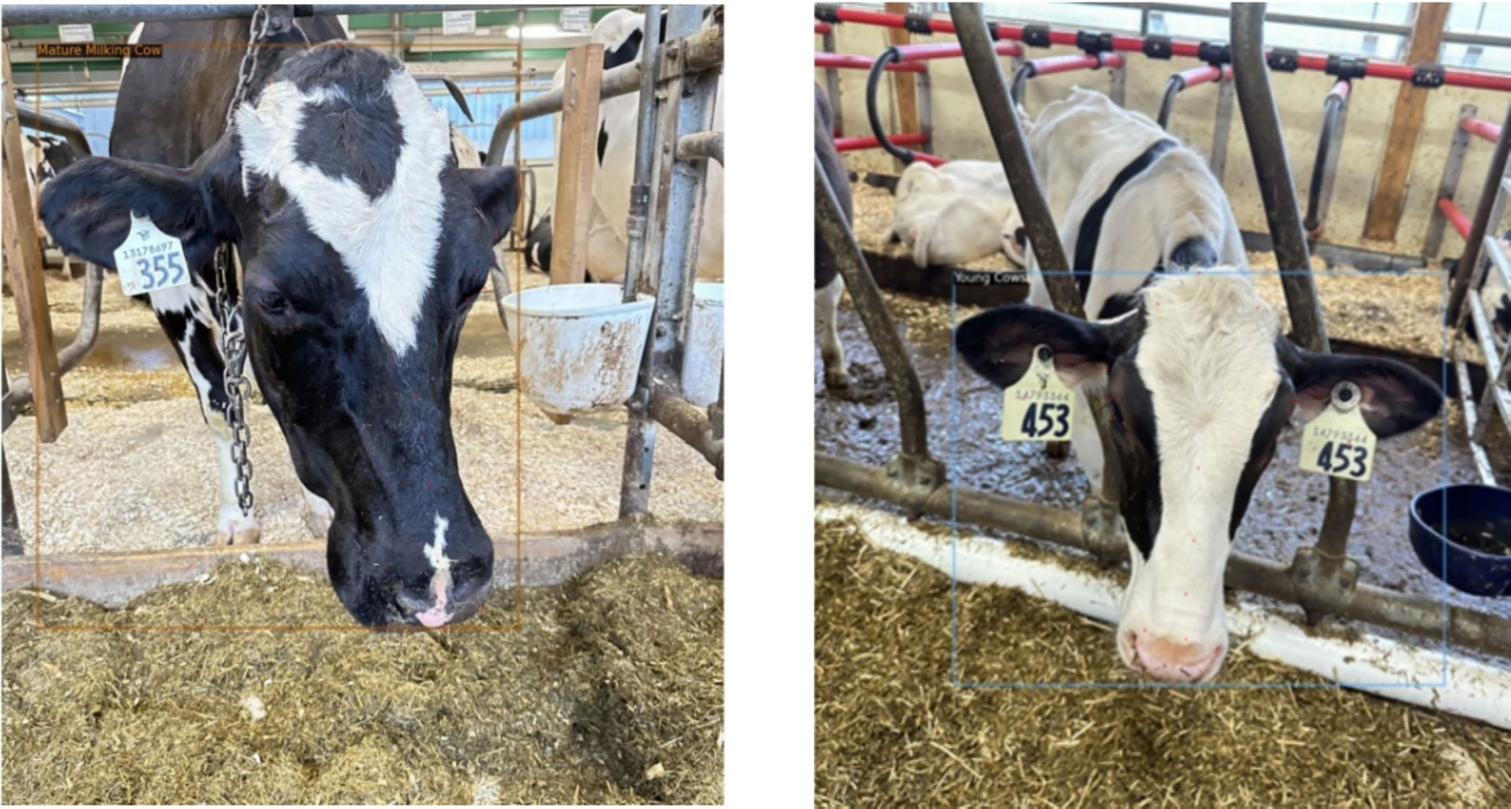
Representative output from the dairy DigiD system showing cattle facial images annotated with bounding boxes, predicted classification labels, and 30 keypoints. This visualization highlights Detectron2’s ability to perform detailed facial biometric analysis by accurately localizing anatomical landmarks and associating them with physiological category predictions under real-world farm conditions.

Detectron2’s architecture (Figure 3) comprises a ResNet-50 backbone with Feature Pyramid Networks (FPN) for multi-scale feature extraction (He et al., 2016; Lin et al., 2017). A Region Proposal Network (RPN) identifies candidate object regions, which are then passed through ROI heads responsible for classification, bounding box regression (Figure 4), segmentation, and keypoint detection. ROI Align preserves spatial resolution during feature pooling, ensuring precise alignment critical for landmark localization.

#### 3.4.2 Multi-task loss and symmetry augmentation

Detectron2 employs a multi-task loss function that combines classification (cross-entropy), bounding box regression (smooth L1), segmentation (binary cross-entropy), and keypoint detection losses. The keypoint loss is computed either via heatmap-based cross-entropy or smooth L1 regression, with optional weighting via Object Keypoint Similarity (OKS). The ROI head was configured to evaluate 128 proposals per image. A custom keypoint flip map was implemented to preserve left–right anatomical symmetry during training, supporting consistent keypoint labeling across mirrored poses.

### 3.5 Robustness in real-world farm conditions

Detectron2 exhibited strong performance under real-world farm conditions, handling occlusions, lighting variations, and cluttered scenes effectively. Detectron2’s robustness under varied environmental conditions aligns with its demonstrated performance in other challenging domains, where it has been shown to effectively localize targets in visually complex scenes (Abdusalomov et al., 2023). Integration with high-resolution video frames, along with landmark-based constraints, enhanced its adaptability. However, training required careful resource management due to GPU memory constraints and CUDA compatibility issues. Variations in cattle appearance—such as breed-specific features, ear positioning, and coat patterns—posed classification challenges, which were mitigated by the anatomical focus of the model and the structural consistency offered by landmark-based detection. Similar challenges were addressed by Xiao et al. (2022), who combined an improved Mask R-CNN with SVMs to achieve cow identification in free-stall barns, highlighting the need for segmentation-aware models in practical deployments.

## 4 Results

To assess the performance of the investigated Detectron2 and DenseNet121 models in classifying dairy cattle, we systematically evaluated each approach’s capacity to accurately differentiate among four distinct categories: Young Cows, Dry Cows, Mature Milking Cows, and Pregnant Cows. The analysis concentrated on standard evaluation metrics precision, recall, and F1-score, alongside convergence analysis to establish model robustness.

### 4.1 DenseNet121 baseline performance

#### 4.1.1 Performance and limitations

The DenseNet121 model achieved strong performance, recording a training accuracy of 98.1% and a test accuracy of 97.1%. However, classification performance was uneven across categories.

The evaluation on the test dataset reveals strong overall classification performance across all cow categories. Notably, Young Cows and Pregnant Cows achieved perfect F1-scores of 1.00, indicating highly accurate and consistent predictions for these classes. *Mature Milking Cows* also showed robust performance with a high F1-score of 0.96, supported by the largest sample size. While *Dry Cows* exhibited slightly lower recall (0.91), their precision remained high (0.97), resulting in a respectable F1-score of 0.94. These results suggest the model is particularly effective in distinguishing between distinct physiological states, though minor misclassifications may still occur in closely related categories such as *Dry Cows*.

#### 4.1.2 Attention mapping and diagnostic insights

**Figure 5:**
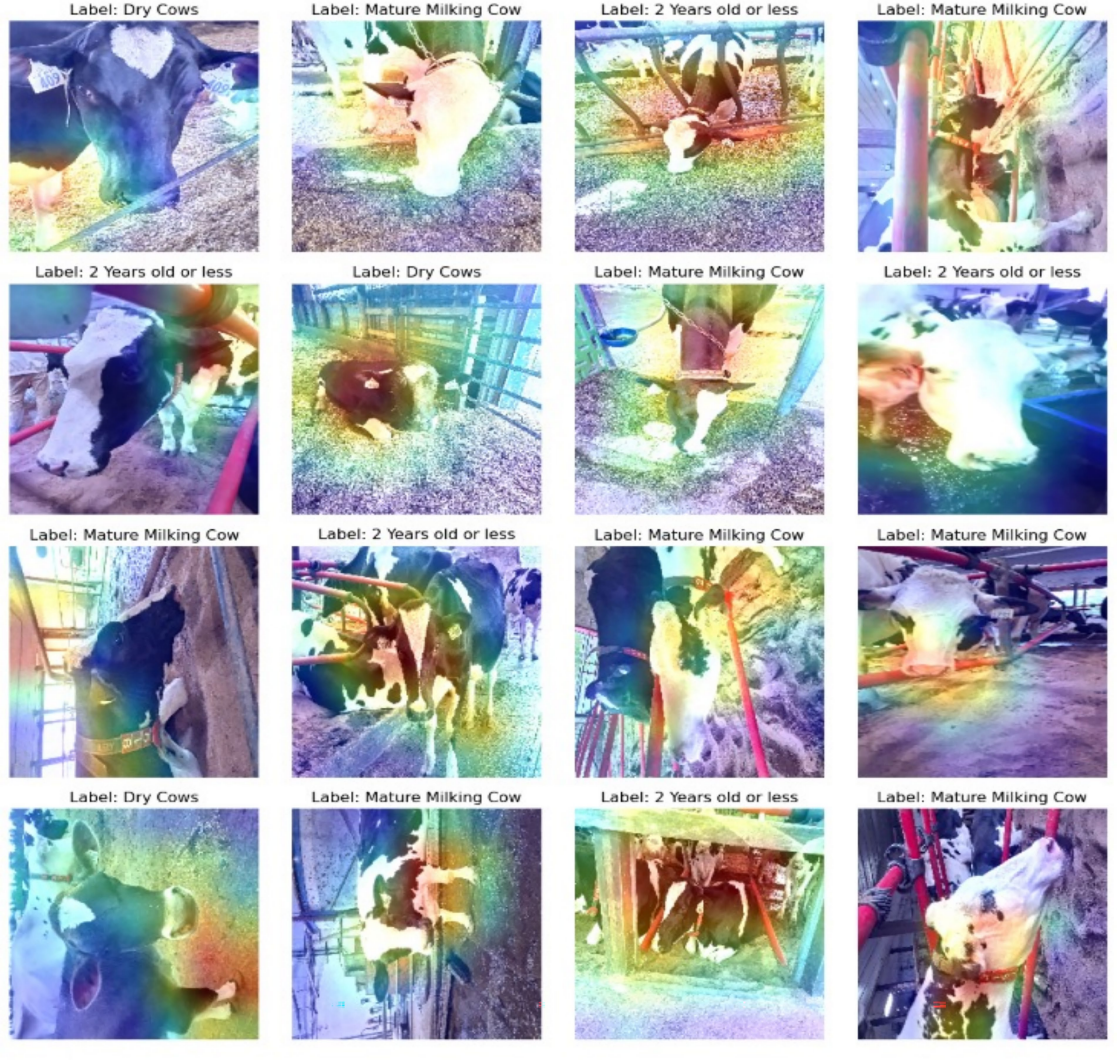
Grad-CAM visualizations highlighting DenseNet121’s attention regions during classification. Heatmaps show that predictions were not consistently driven by facial features, with attention sometimes misdirected to background or body areas, thereby motivating a shift to a facial landmark-based approach.

To examine the internal logic of DenseNet121’s predictions, we applied Gradient-weighted Class Activation Mapping (Grad-CAM). As shown in Figure 5, while the model occasionally attended to informative facial areas, it frequently focused on irrelevant regions such as the neck, legs, or barn backgrounds. These inconsistencies underscored the need for a more constrained approach that focuses explicitly on biologically salient facial features.

Visual overlap between “Young” and “Pregnant” cows led to misclassifications, suggesting that the model relied on global features like posture, ear shape, and coat color, which were contextually variable and not always discriminative. This reliance reduced generalization performance, especially under changing environmental conditions.

### 4.2 Overall performance of Detectron2

The Detectron2 framework demonstrated robust overall classification performance on the independent test set. After training, the model achieved an overall weighted accuracy of 0.93 and a weighted average F1-score of 0.92 (Table 1 for detailed per-class metrics on the test set).

**Table 1:** Five-fold cross-validation results illustrating fold-wise accuracy and F1-score, along with mean values and standard deviations, highlighting the Detectron2 model’s robustness and capacity for consistent generalization across diverse data partitions.

Fold	Accuracy	F1-score
1	0.7867	0.7602
2	0.8252	0.8176
3	0.7587	0.7456
4	0.7902	0.7845
5	0.7965	0.7711
Mean	0.7915	0.7758
Std	0.0197	0.0283

### 4.3 Training convergence and validation stability

**Figure 6:**
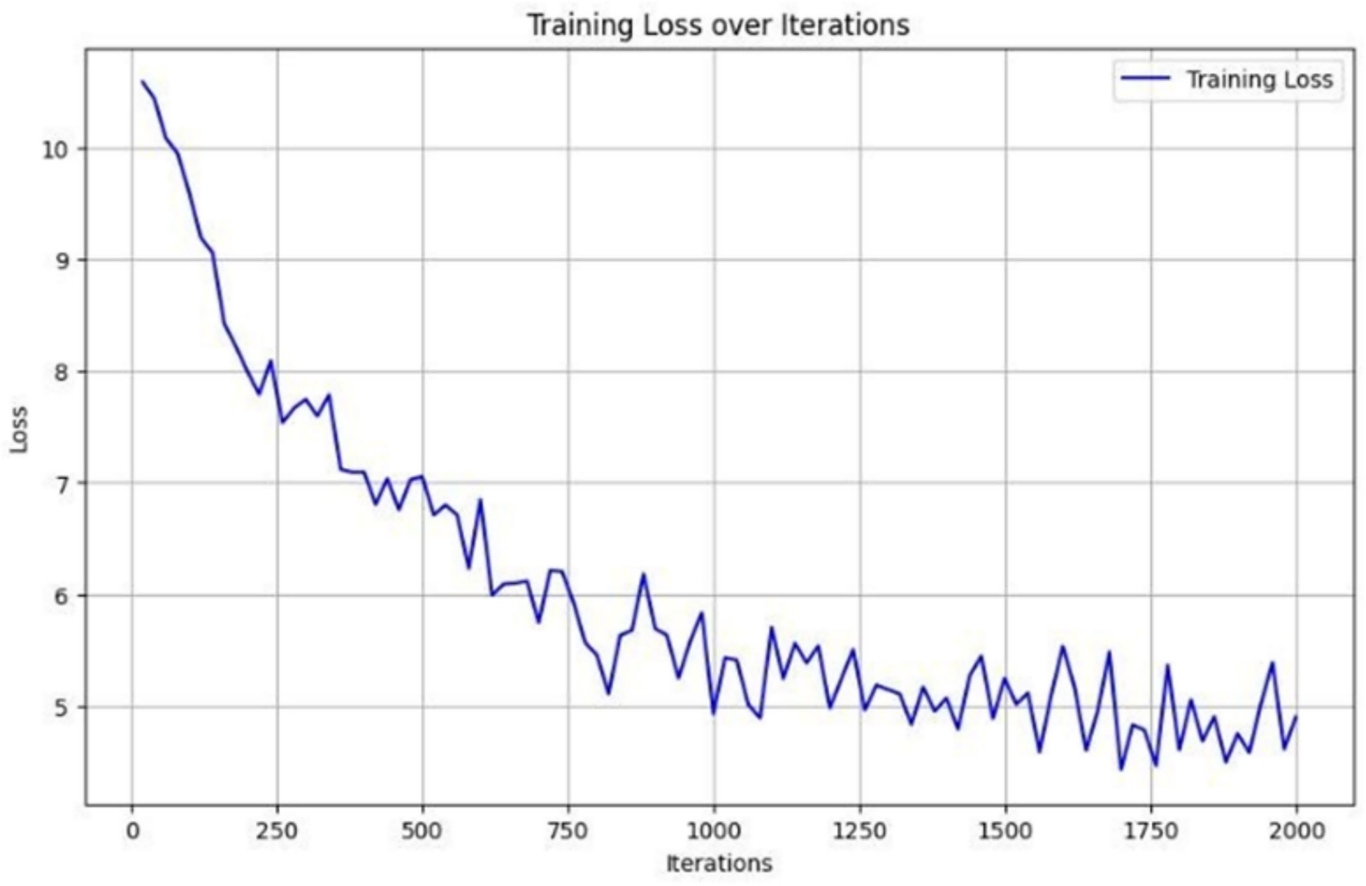
Training loss curve of the Detectron2 model across 2,000 iterations, demonstrating rapid initial learning followed by gradual convergence, indicating effective feature extraction and stable optimization.

**Figure 7:**
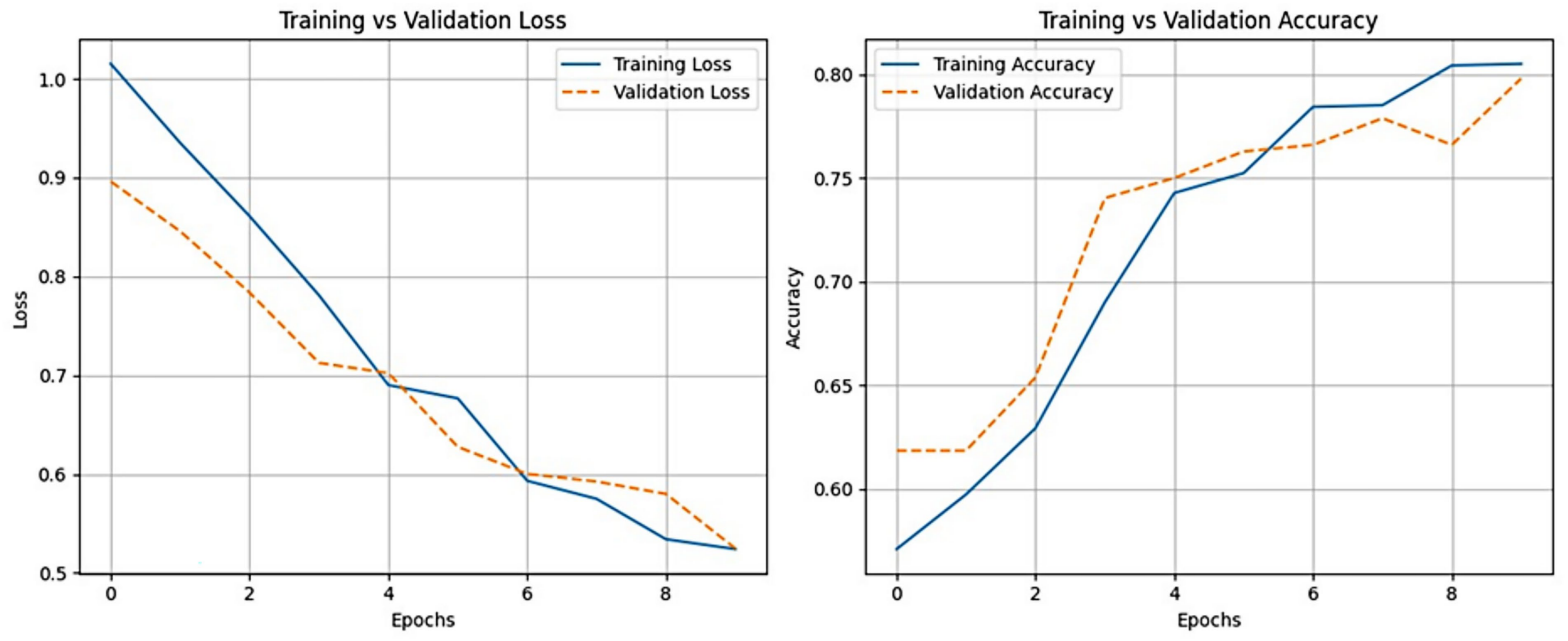
Training and validation loss (left) and accuracy (right) across epochs for the Detectron2 model, confirming consistent convergence and minimal overfitting.

We closely monitored Detectron2’s training performance across 2,000 iterations. As shown in Figure 6, the model exhibited three distinct training phases. In the initial phase (0–250 iterations), a sharp reduction in loss indicated rapid learning of basic visual features. During the intermediate phase (250–1,500 iterations), the model fine-tuned class-distinguishing features, and in the final phase (after 1,500 iterations), the loss curve plateaued, signaling convergence. Further validation results are illustrated in Figure 7. Here, training and validation loss and accuracy are plotted per epoch. The close alignment between validation and training curves supports strong generalization. The model showed no major signs of overfitting across epochs, aided by early stopping protocols and regularized training.

### 4.4 Environmental factors and generalization performance

The performance of the Detectron2 model was shaped not only by its architecture but also by environmental conditions and data partitioning strategies. Similar effects have been documented by Chen et al. (2021), who reported a decline in model performance when cattle were imaged under heterogeneous farm conditions. To quantify generalization, we conducted a 5-fold cross-validation using the training and validation subset associated with the Detectron2 model. Each fold maintained an 80-to-20 split between training and validation data, with stratified sampling to preserve class proportions.

These results confirm the model’s consistent generalization capability across diverse data subsets, highlighting stability and minimizing concerns of overfitting within the cross-validation process.

### 4.5 Addressing class imbalance with ROC-AUC analysis

**Figure 8:**
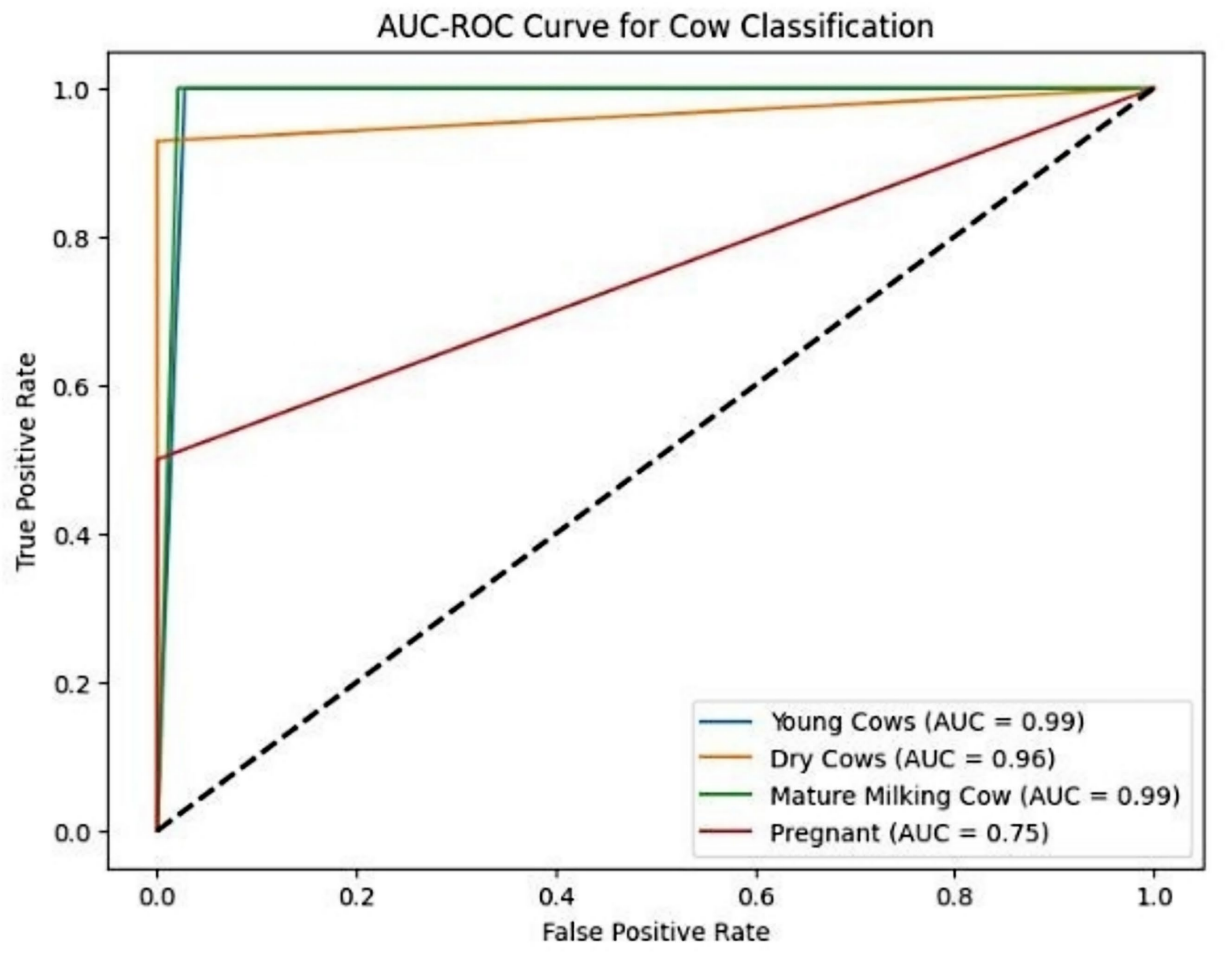
Multi-class ROC curves illustrating the Detectron2 model’s discriminative performance. The “Pregnant Cow” class exhibited reduced separability, reflecting class imbalance and visual overlap.

To better understand the effect of class imbalance, we conducted ROC-AUC analysis (Figure 8). The “Young Cow” and “Mature Milking Cow” classes displayed exceptionally high separability with AUC values of 0.99, while the “Pregnant Cow” class showed a notably lower AUC of 0.75. While the “Pregnant Cow” class exhibited a lower AUC of 0.75, this level of performance is still indicative of effective discrimination, especially given the visual similarity with other classes and the inherent biological challenges of this task. Such challenges are not unexpected in livestock classification tasks, where closely related physiological categories often exhibit subtle anatomical differences that make clear separation more difficult.

### 4.6 Explainability via grad-CAM and perturbation-based analyses

**Figure 9:**
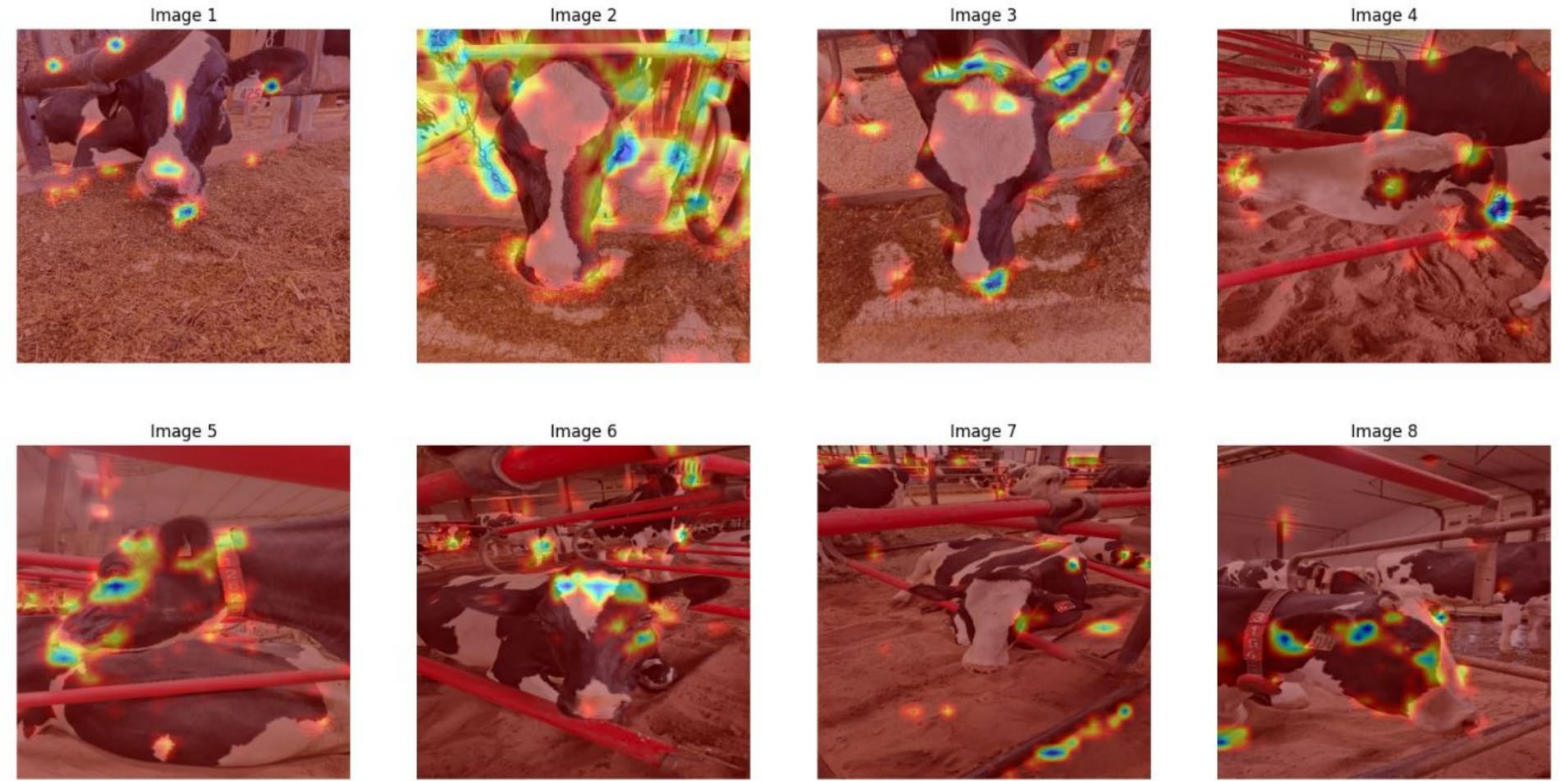
Gradient-weighted class activation mapping (Grad-CAM) visualizations for Detectron2, highlighting biologically relevant attention regions.

**Figure 10:**
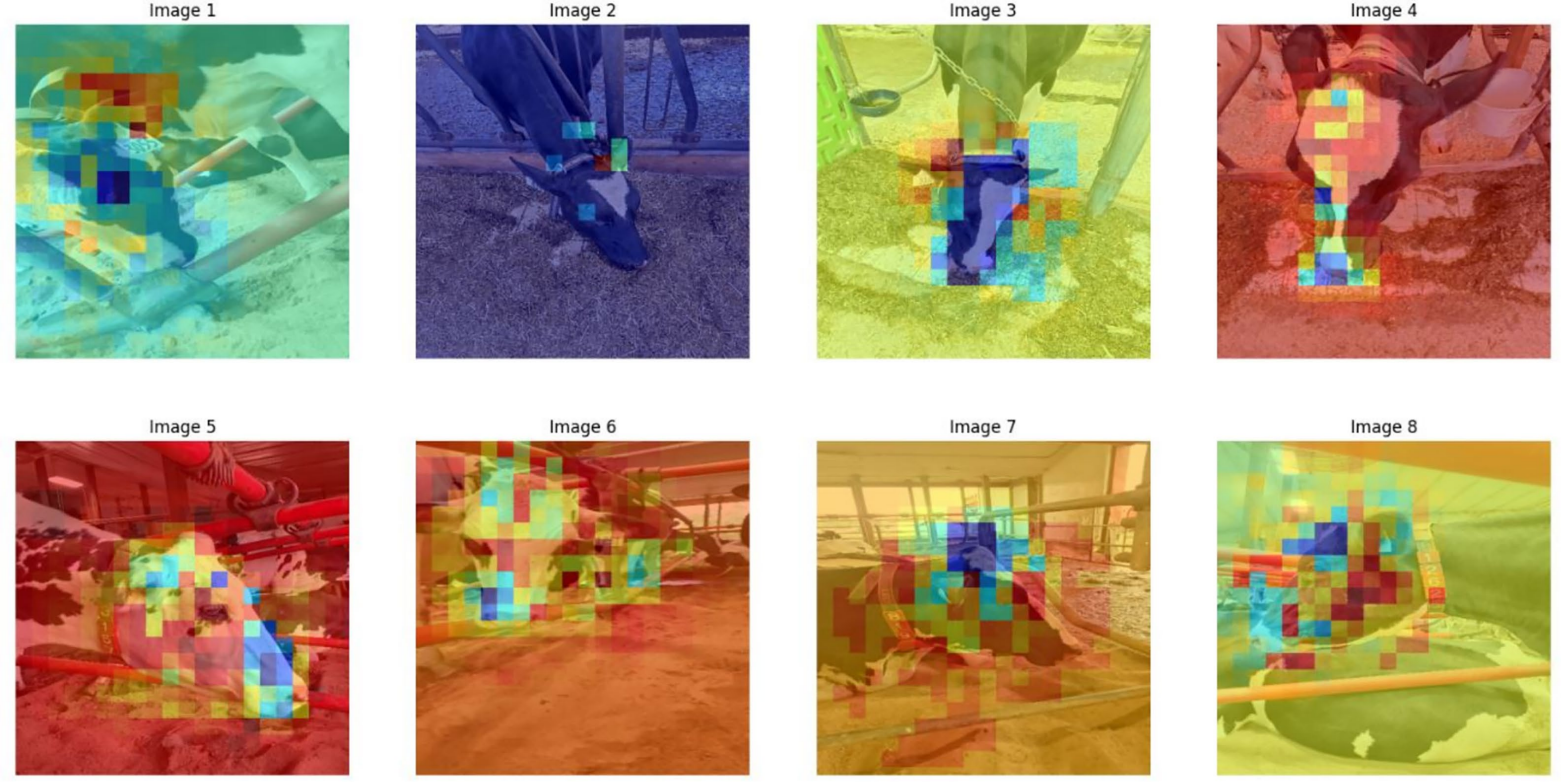
Perturbation-based explainability heatmaps revealing sensitivity to occlusions of key facial landmarks like the nostrils and eyes.

Grad-CAM visualizations (Figure 9) validated that Detectron2’s predictions were grounded in meaningful facial regions. Keypoints such as the nostrils, eye center, and muzzle perimeter were highly activated, reflecting alignment with biologically relevant landmarks. To test robustness, we employed Perturbation-Based Explainability (PBE), systematically occluding facial features and observing the effect on prediction confidence. Figure 10 shows that occlusions of central features (e.g., muzzle or eye region) caused significant performance drops. Occluding peripheral or non-facial regions had little effect, reinforcing the critical role of facial landmarks.

The results indicate that both models deliver high classification performance across most physiological categories. Detectron2 demonstrated robust precision and recall, with especially strong separation between classes, which may reflect the impact of its keypoint-based architecture. DenseNet121 achieved marginally higher F1-scores in most categories on the independent test set, except for Dry Cows, where its F1-score was slightly lower than that of Detectron2. These findings suggest that both global (DenseNet121) and landmark-driven (Detectron2) approaches are effective for visual cattle classification, with nuanced performance differences across specific categories.

### 4.7 Saliency analysis via grad-CAM

To better understand the basis of model decision-making, we examined saliency maps generated by Grad-CAM for both Detectron2 and DenseNet121. These visualizations indicate which image regions most strongly influenced the models’ classifications. For Detectron2, Grad-CAM activations were consistently concentrated on specific facial features—particularly the predefined anatomical landmarks of the muzzle and eyes—with a majority of activation mass (~80%) localized in these relevant regions across diverse test images. In contrast, DenseNet121 exhibited more diffuse attention, with a substantial proportion of its activation extending to background elements such as barn rails and bedding; only around 45–55% of the activation typically fell on the animal’s face. These differences suggest the keypoint-based model’s architecture is more anatomically focused and potentially less susceptible to background confounding.

### 4.8 Generalization and comparative performance on the independent test set

To assess the real-world utility of our models, we evaluated both Detectron2 (keypoint-based) and DenseNet121 (full-image classifier) on a shared, independent test dataset representing all four physiological cattle categories. Table 2 summarizes classification metrics—precision, recall, and F1-score—for both the models and class. Detectron2 demonstrated robust generalization, with strong performance on “Dry Cows” and “Young Cows.” “Pregnant Cows” were identified with high recall and improved F1-score, reflecting the benefits of targeted data augmentation and fine-grained anatomical feature use. For “Mature Milking Cows,” Detectron2 achieved perfect precision but somewhat lower recall, attributable to class imbalance in the test set.

**Table 2:** Classification metrics (precision, recall, and F1-score) for Detectron2 and Densenet121 on the independent test set.

Model	Detectron2			Densenet121		
Category	Precision	Recall	F1-Score	Precision	Recall	F1-Score
Young cows	1.00	0.93	0.96	0.99	1.00	1.00
Dry cows	0.98	1.00	0.99	0.97	0.91	0.94
Mature milking cows	1.00	0.65	0.67	0.95	0.98	0.96
Pregnant cows	0.94	1.00	0.97	1.00	1.00	1.00

Direct comparison of Detectron2 and DenseNet121 was conducted using standardized confusion matrices and the same evaluation metrics. This side-by-side analysis revealed that while both models performed well across most categories, Detectron2 generally provided tighter class separation and greater sensitivity to group-specific anatomical features, whereas DenseNet121—trained on holistic visual patterns—exhibited higher sensitivity to background variation and greater variability in misclassification patterns. These results indicate the complementary strengths of anatomical keypoint and full-image strategies for automated cattle classification.

## 5 Discussion

This study provides a comprehensive assessment of two deep learning frameworks—DenseNet121 and Detectron2—for non-invasive dairy cattle classification. The results highlight both progress and remaining limitations in deploying automated vision systems under field conditions. Our findings show that while both models achieve strong overall performance, the keypoint-based Detectron2 framework offers significant advantages in robustness and interpretability by focusing on anatomically consistent facial features rather than variable full-image context.

### 5.1 The role of class imbalance in model performance

**Figure 11:**
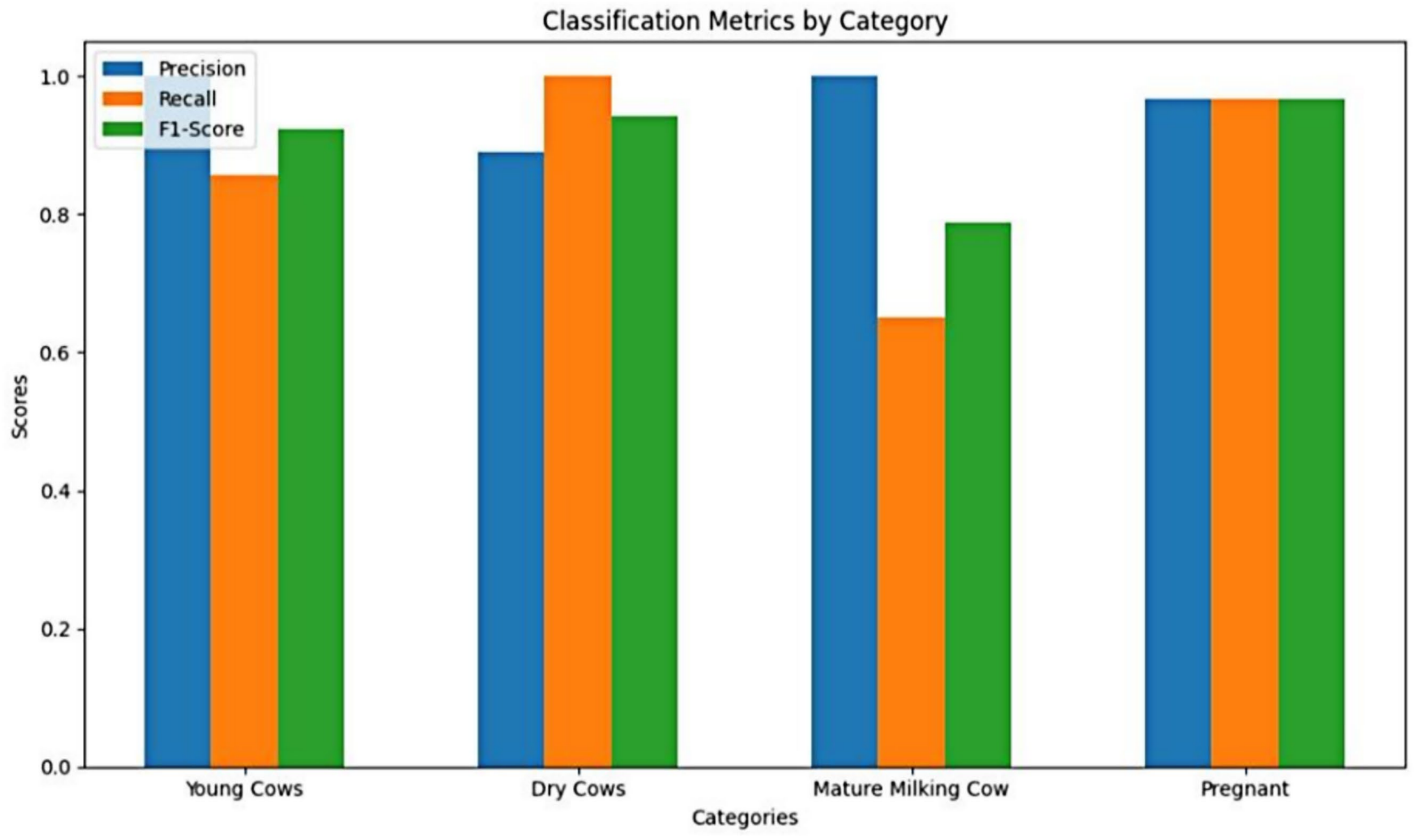
Comparative bar chart of precision, recall, and F1-score for each cattle category. Lower recall for “Mature Milking Cows” reflects small test sample size.

**Figure 12:**
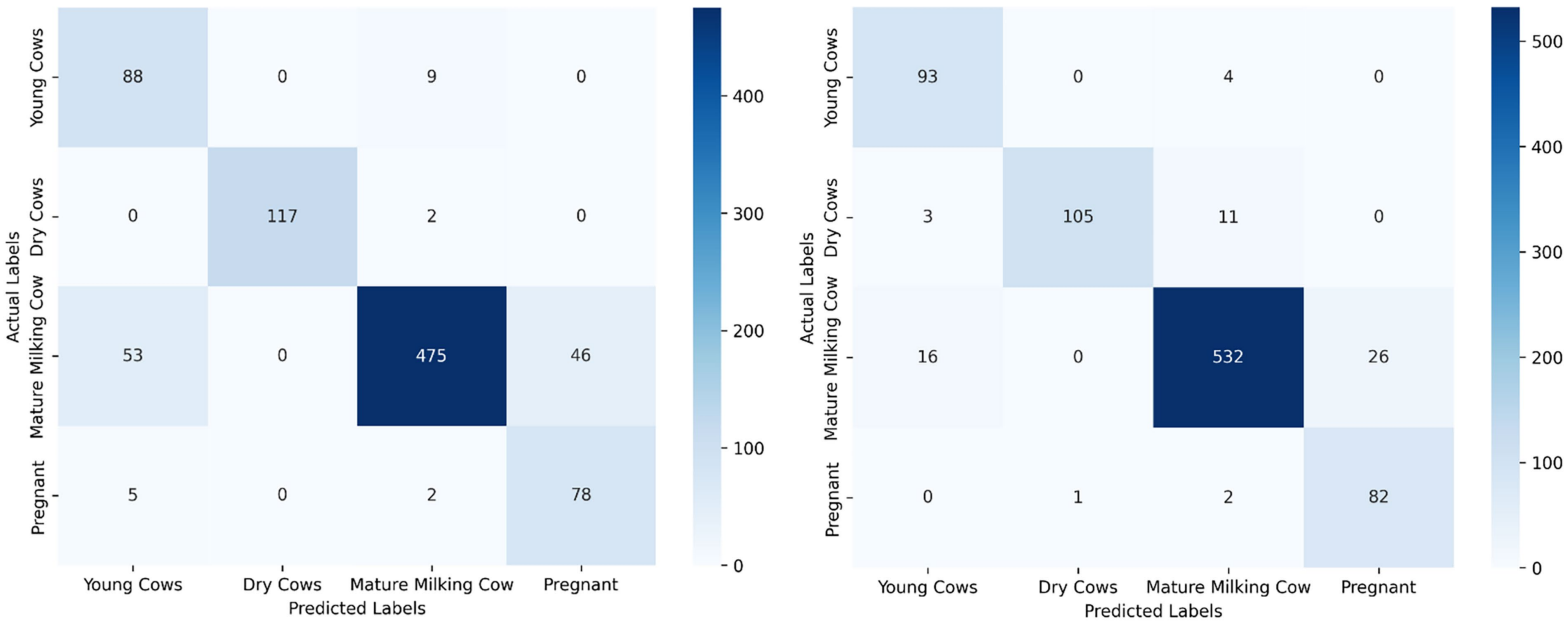
Side-by-side confusion matrices for Detectron2 (left) and DenseNet121 (right), showing that DenseNet121 is more sensitive to background noise, while Detectron2 offers tighter class separation.

One of the persistent challenges faced was class imbalance, stemming primarily from unequal representation across categories in the dataset. The relatively lower performance of the Detectron2 model on the “Pregnant Cow” class—even with targeted augmentation—can be directly attributed to this limited representation during training. As noted by Hossain et al. (2022), underrepresented categories in livestock datasets often yield reduced accuracy due to less learning exposure, a trend further corroborated by Li et al. (2022) in their study of cattle recognition systems under dataset skew. This pattern is evident in our results: ROC-AUC analysis (Figure 8) and per-class metrics (Table 2) both illustrate the effect of class imbalance. Despite these challenges, category-level analysis reveals strong overall model performance; for example, “Mature Milking Cow” achieved perfect precision (1.00) on the test set, while “Young Cows” returned a high F1-score of 0.96 and “Pregnant Cows” reached an F1-score of 0.97 (Table 2 and Figure 11). The “Pregnant Cow” category, represented by just 89 images before augmentation, registered a lower AUC (0.75) and showed greater confusion with visually similar groups such as “Young Cows” (Figure 12), leading to reduced classification metrics in some evaluation phases.

Despite being the majority class with extensive representation in the dataset, the “Mature Milking Cow” category exhibited lower recall (0.65) and F1-score (0.67) in Detectron2, suggesting that factors beyond sample size—such as higher intra-class variability or feature overlap with other groups—may have limited the model’s class-specific generalization. In contrast, “Young Cows” and “Dry Cows” showed more consistent and robust classification results across evaluation metrics (Table 2). Models trained on imbalanced datasets typically bias predictions toward majority classes, limiting their capacity to accurately generalize features of minority categories. These findings indicate that even state-of-the-art models like Detectron2 struggle to learn discriminative representations when trained on imbalanced datasets.

Although the 300-image calibration round (*κ* = 0.87) greatly reduced inconsistencies, residual label noise in the full corpus can still propagate systematic bias—capping attainable F1 scores and magnifying errors for under-represented classes. Duplicating all 8,700 images was financially prohibitive in the present study, so future work will (i) compute a corpus-wide Fleiss’ κ, (ii) train with noise-robust losses such as generalized cross-entropy (Zhang and Sabuncu, 2018), and (iii) deploy an active-learning relabelling loop that automatically surfaces uncertain samples for expert review, progressively purging noise and tightening performance estimates. To further reduce minority-class error, we will generate high-quality synthetic images via class-conditional GANs and expand targeted data collection, injecting realistic intra-class variability without prohibitive annotation costs (Mullick et al., 2019).

### 5.2 Transition to keypoint-based classification

The anticipated performance limitations of DenseNet121, particularly its sensitivity to background noise and contextual variability, motivated a transition to Detectron2. Detectron2 offers a modular framework for object detection, instance segmentation, and keypoint detection (Wu et al., 2019). By focusing on detailed facial features, it enables classification based on structurally stable, pose-invariant anatomical landmarks. This approach was hypothesized to be more beneficial in farm environments characterized by clutter, occlusion, and lighting inconsistencies. The new pipeline emphasized robustness and interpretability by anchoring the model’s decision-making to consistent facial biometric indicators.

### 5.3 Visual overlap and the limits of facial biometrics

Our findings reaffirm that fine-grained classification between physiologically adjacent categories remains a challenge, especially when using facial biometrics alone. Facial landmarks like eyes, ears, nostrils, and muzzle generally offer stable biometric references less prone to short-term variability. Detectron2’s reliance on 30 anatomical landmarks—although robust to pose and lighting variation—was not always sufficient to resolve inter-class ambiguities between “Young” and “Pregnant” cows or “Pregnant” and “Mature Milking” cows, suggesting the intrinsic limitations of purely landmark-focused classification when subtle physiological and phenotypical differences are present. Similar challenges have been observed even in models employing sophisticated face recognition pipelines such as CattleFaceNet, which combined RetinaFace detection with ArcFace embeddings for cattle identification (Xu et al., 2022). This issue has been echoed in prior work by Zhang et al. (2023a, 2023b), who observed similar limitations in sheep recognition, where subtle anatomical variation led to poor CNN differentiation. Comparable challenges have also been noted in cattle face recognition systems leveraging parameter transfer and deep learning, where high intra-class similarity and subtle phenotypic differences can limit classification robustness (Wang et al., 2020).

In contrast, DenseNet121, which processes the full image, often leveraged environmental features such as background context or body posture. While this broader view sometimes helped with class separation, it also increased sensitivity to irrelevant visual noise, as shown by our Grad-CAM results (Figure 5). These observations support Qiao et al. (2021), who advocate for incorporating multimodal cues—including behavioral indicators, skeletal structure, and body condition metrics—to improve discriminatory power. Aligns with broader trends in precision livestock farming, where machine learning models are increasingly employed to analyze not only visual but also temporal behavioral data, such as feeding patterns, to optimize animal management (El Moutaouakil and Falih, 2024). This observation highlights the potential benefit of integrating complementary information such as body posture, coat condition, or environmental context to enhance discrimination accuracy. Going forward, a promising avenue would be to fuse facial biometric inputs with other data modalities (e.g., skeletal keypoints, movement data, or even thermal patterns) to enable more robust classification, particularly between adjacent physiological states (Neethirajan and Kemp, 2021).

### 5.4 Environmental conditions and image quality effects

As demonstrated in Sections 4.5 and 4.9, model performance was significantly influenced by environmental and imaging conditions. Images with consistent lighting, clean backgrounds, and frontal views (e.g., many “Dry Cow” and “Young Cow” images) yielded the highest precision, recall, and F1-scores. In contrast, “Pregnant Cow” images, which were captured under a variety of lighting and pose conditions, led to higher misclassification rates and reduced recall. Variability resulting from environmental factors such as sunlight glare, shadows, or partial occlusions by farm infrastructure or other animals increased classification errors and decreased model reliability. Images captured with consistent lighting, clear frontal facial orientation, and minimal background clutter were associated with the most robust classification outcomes. Notably, the “Dry Cow” and “Young Cow” categories—comprising a larger proportion of such high-quality images—achieved the most reliable results across all evaluation metrics. By contrast, the “Pregnant Cow” category exhibited a higher rate of misclassification, attributable to both its lower representation and greater variability in imaging conditions, including shadowing, reflective glare, partial occlusions, and off-angle viewpoints.

These findings align with Chen et al. (2021), who observed that heterogeneous visual backgrounds decreased recognition performance in Angus cattle. While data augmentation strategies—including contrast enhancement, rotation, and noise injection—were implemented to simulate real-world variation, augmentation alone could not completely close the domain gap. These observations underscore the critical need for standardized data collection protocols and robust augmentation techniques designed to impart invariance to environmental variations, strengthening the model’s resilience in practical deployments.

Environmental conditions also contributed to performance variability across folds. In some subsets, images included adverse visual features such as barn fixtures, backlighting, or motion blur, all of which increased classification error. While data augmentation techniques such as brightness modulation, random contrast shifts, and geometric transformations were employed to improve robustness, they were insufficient to fully replicate the complexity of real-world farm imagery.

These findings underscore the importance of addressing both dataset imbalance and environmental heterogeneity. Enhancing the consistency of image capture through standardized protocols, and expanding augmentation strategies to include more adaptive techniques such as style-transfer or domain-aware photometric transformations, may substantially improve the model’s capacity to generalize to novel conditions. Such refinements are essential for ensuring that cattle classification systems perform reliably across the diverse operational settings encountered in commercial dairy production.

As a result, establishing image collection guidelines (e.g., preferred angle, illumination, and background) will be key to improving future system deployments. Moreover, advanced augmentation methods such as style-transfer or domain-adaptive GANs may allow for improved simulation of diverse environmental conditions, further strengthening model generalization.

### 5.5 Generalization and model tuning behavior

Our cross-validation results (Table 2) and convergence plots (Figures 6, 7) indicate that Detectron2 demonstrated consistent learning behavior and good generalization to unseen validation data. However, performance on the held-out independent test set was significantly higher (weighted F1-score ~0.92) than average cross-validation scores (F1-score ~0.7758). This discrepancy reflects variation in environmental quality across partitions. The independent test set benefited from more uniform lighting and frontal facial views, resulting in stronger model performance. In contrast, the 5-fold validation sets, though stratified, included more heterogeneity. The improved test performance thus affirms the model’s robustness and generalization capacity.

Notably, the “Pregnant Cow” class remained underrepresented even in the cross-validation folds, limiting Detectron2’s exposure during training. The independent test set, by contrast, may have benefitted from better alignment with the augmented training distribution. This finding further reinforces the impact of class imbalance and motivates targeted data balancing during fold generation. Focal loss and other class-aware strategies partially mitigated this problem, but did not fully resolve it. Empirical tuning showed that 10 epochs were sufficient for convergence, with validation loss showing minimal improvement beyond 8–9 epochs. Hence, both convergence dynamics and augmentation strategies should be carefully aligned with the specific challenges of class skew and real-world variation. Complementary to these strategies, software-level testing frameworks have been proposed to evaluate the internal consistency of deep learning models in image analysis tasks, which could further enhance reliability in agricultural deployments (Tian et al., 2019). This conclusion was based on real-time monitoring of loss curves, as shown in Figures 6, 7, where the training and validation loss trajectories stabilized before reaching the 10th epoch. To operationalize this, an early stopping protocol was implemented with a patience threshold of 2 epochs, further safeguarding against overfitting. This ensured that training was halted if no meaningful improvement in validation loss was observed. Thus, the choice of 10 epochs served as an upper bound rather than a fixed target, and final epoch counts varied slightly depending on convergence behavior within each fold.

### 5.6 Addressing model overfitting and enhancing generalizability

Although overfitting was not a prominent concern in our study (Figures 6, 7), real-world deployment of models trained in academic settings can face challenges related to dataset representativeness. We used cross-validation, diverse farm environments (Nova Scotia and New Brunswick), and data augmentation to mitigate this risk. Still, further generalizability could be achieved by testing geographically diverse datasets involving different breeds, camera setups, and housing systems. Qiao et al. (2021) similarly calls for expanded validation across operational contexts to ensure deployment readiness. Thus, future benchmarking studies should incorporate a wide range of geographic and management system variation to produce broadly applicable models.

### 5.7 Real-time deployment and scalability

For deployment, Detectron2 inference time (~50–60 ms per image, ~30 FPS) on an NVIDIA Tesla T4 GPU was promising. These speeds are sufficient for real-time implementation in structured environments such as milking parlors or feeding stations. To scale further, model compression techniques like quantization and pruning can be applied to reduce latency and memory usage, as has been done successfully in other object detection domains (Lyu et al., 2024). Additionally, integrating Detectron2’s output with herd management systems (e.g., DairyComp 305) using REST APIs could allow farms to receive real-time alerts on animal status. By situating fixed cameras in high-traffic farm zones, automated cattle monitoring can be realized without disrupting daily routines. Our preliminary tests showed that selective pruning reduced memory demands by ~40% with negligible performance loss, supporting scalability in resource-constrained environments.

### 5.8 Interpretability and practical explainability

Interpretability is a key requirement for building trust in AI-powered livestock monitoring. Explainable AI techniques not only improve model transparency but also enable biological interpretation, where phenotypic differences were linked to AI-detected patterns (Carrieri et al., 2021). We adopted Grad-CAM and perturbation-based explainability, which are more suited for object detection pipelines. Grad-CAM heatmaps (Figure 11) confirmed that model predictions were based on biologically relevant landmarks such as the nostrils, eyes, and muzzle. Perturbation-Based Explainability (Figure 12) reinforced this, showing performance degradation when keypoints were masked. While this study utilizes Grad-CAM and perturbation-based explainability tailored for deep visual models, other approaches like SHAP and LIME offer model-agnostic feature attribution in structured data contexts, although their integration with complex vision architectures remains limited (Chen et al., 2022; Salih et al., 2025). These tools not only confirm model validity but also offer actionable feedback for data collection and model refinement. For instance, if occlusions in specific facial zones cause sharp drops in performance, targeted data augmentation or occlusion-aware training can be introduced.

### 5.9 Comparative interpretations

To directly compare the performance of the keypoint-based Detectron2 model with the full-image DenseNet121 classifier, we evaluated both approaches on the independent test set. The analysis focused on confusion matrices and standard classification metrics (precision, recall, and F1-score) for each cattle category.

The Grad-CAM-based saliency analysis offers insight into each model’s interpretability and robustness. Detectron2’s attention to face-specific landmarks aligns with its keypoint-based design, likely contributing to its resilience under real-world imaging conditions that include variable backgrounds and occlusions. This anatomically consistent focus enhances transparency and trust in automated decision-making. Conversely, the broader and sometimes background-driven activations observed in DenseNet121 suggest potential vulnerability to contextual noise and less interpretability, despite its competitive performance on some test categories. These findings emphasize that explicit anatomical modeling—not just raw predictive accuracy—plays an important role in building dependable livestock biometric systems. Future work should further quantify and leverage attention patterns to improve both performance and explainability.

Whereas a direct comparison of classification metrics reveals distinct performance patterns between the Detectron2 and DenseNet121 models (Tables 1, 2). DenseNet121 achieved consistently high precision and recall across all categories, with perfect F1-scores of 1.00 for Young and Pregnant Cows, and robust performance for Mature Milking Cows (F1 = 0.96) and Dry Cows (F1 = 0.94). In contrast, Detectron2 exhibited superior precision for categories such as Young Cows (1.00) and Mature Milking Cows (1.00), but showed a notably reduced recall (0.65) for Mature Milking Cows, resulting in a lower F1-score (0.67). However, Detectron2 demonstrated excellent balance for Dry Cows (F1 = 0.99) and Pregnant Cows (F1 = 0.97), outperforming DenseNet121 in these specific groups. These differences underscore how DenseNet121’s reliance on global morphological cues provided more uniform sensitivity across classes, whereas Detectron2’s keypoint-based strategy enhanced specificity but at the cost of recall in certain physiologically similar categories. This trade-off highlights the importance of aligning model choice with operational priorities whether minimizing false negatives (favoring recall) or ensuring high-confidence positive identifications (favoring precision).

### 5.10 Future directions: architecture and dataset innovation

Looking ahead, improving classification performance requires more than refining CNN architectures. Vision Transformers (ViTs) with their ability to capture long-range dependencies and multi-scale context, show promise for resolving ambiguities between classes with overlapping facial morphology (Zhang et al., 2023a, 2023b). A hybrid CNN-ViT pipeline could offer improved feature localization and global context integration. While RGB imaging remains the foundation for most livestock classification systems, future work should leverage multi-modal benchmarks such as CattleFace-RGBT, which combine RGB with thermal imaging and standardized facial landmarks to enhance generalization and physiological insight (Coffman et al., 2024).

From a dataset perspective, the priority should be increasing class balance and anatomical diversity through synthetic data, targeted collection, and multimodal integration. Beyond RGB images, modalities such as 3D facial scans, thermal imaging, or audio recordings of vocalizations may yield richer, complementary signals (Abdrakhmanova et al., 2021). These innovations, combined with adaptive loss functions (e.g., focal loss, class-weighted cross-entropy), will drive further gains in both classification performance and interpretability.

### 5.11 Ethical and societal implications

As AI becomes more embedded in livestock operations, ethical considerations grow increasingly important. Systems like Dairy DigiD must be accompanied by clear protocols for data privacy, ownership, and informed consent. Particularly when used on commercial farms, data sharing agreements must ensure that biometric data is not repurposed or used without stakeholder oversight (Neethirajan, 2023a, 2023b). Broader concerns about the social acceptability of facial recognition systems have also been highlighted in user studies (Seng et al., 2021), underscoring the importance of transparent consent processes and clear communication about data use. As Hagendorff (2020) cautions, many AI ethics frameworks risk becoming symbolic unless grounded in actionable principles. This is echoed in the broader agricultural AI literature, where Ryan (2023) highlights the need for more tangible, socially responsive approaches to deploying AI technologies on farms. In this context, Dairy DigiD aligns ethical intent with practical design by emphasizing non-invasive data collection, stakeholder transparency, and animal-centered implementation. Moreover, the risk of digital divide must be addressed. Large operations may be quicker to adopt AI, leaving smaller farms behind due to cost or technical barriers. Policymakers and industry groups must consider strategies for equitable access, such as subsidized hardware, open-source software, and user training programs. The long-term sustainability of AI in agriculture hinges not just on technical performance, but on ethical deployment and inclusive adoption.

## 6 Conclusion

In the evolving landscape of precision livestock farming, the development of accurate, scalable, and ethically aligned systems for cattle categorization is not merely a technical enhancement, but an operational imperative. This study critically examined two deep learning frameworks, DenseNet121 and Detectron2, to evaluate their effectiveness in non-invasive, image-based classification of dairy cattle into physiologically meaningful groups. While both models demonstrated considerable potential, our comparative analysis highlighted important differences in their robustness, interpretability, and practical performance under real-world farming conditions. DenseNet121, used for full-image classification, delivered strong baseline performance by leveraging global morphological and contextual features. However, its reliance on background elements and susceptibility to visual clutter exposed the limitations of generalized convolutional approaches in uncontrolled agricultural environments. In contrast, the keypoint-based Detectron2 model proved more resilient, extracting localized facial landmarks such as the nostrils, eyes, and muzzle to generate anatomically grounded and biologically relevant features. This localized precision enabled more consistent classification even under variations in lighting, camera angle, and environmental noise.

Despite Detectron2’s strong performance, our findings underscore the ongoing challenge of class imbalance, particularly in detecting underrepresented groups such as pregnant cows. Addressing this issue will require improved dataset balance through targeted data augmentation, synthetic image generation using generative adversarial networks, and broader image collection protocols across diverse farm settings. Additionally, the limitations of single-modality input became evident. While facial features offer a stable baseline, combining them with additional data streams such as body posture, behavioral indicators, or thermal imaging could enhance the detection of subtle physiological differences between visually similar categories. Looking forward, the adoption of advanced architectures such as Vision Transformers holds considerable promise. These models can integrate fine-grained spatial detail with broader contextual awareness through self-attention mechanisms, which may improve classification performance for ambiguous cases. When paired with explainability techniques like Grad-CAM or perturbation-based analysis, such architectures could contribute to the development of transparent and trustworthy AI systems for livestock applications.

Ethical considerations must remain central to this advancement. The non-invasive nature of Detectron2 minimizes stress and behavioral disruption, aligning with increasing expectations for animal-friendly technologies and transparent welfare standards. These concerns highlight the importance of human-centric and welfare-conscious AI systems in livestock management (Neethirajan, 2024a, 2024b). Our findings demonstrate that high-performance classification does not have to come at the expense of animal comfort, supporting a shift toward more compassionate, observationally driven farming practices. Ultimately, Dairy DigiD offers more than a technical contribution. It provides a foundation for deploying AI systems that are not only accurate and scalable, but also ethical and context-aware. By integrating cutting-edge deep learning techniques with real-world usability and welfare concerns, this work advances the frontier of precision dairy management and highlights a viable path forward for sustainable, data-driven agriculture.

## Data Availability

The raw data supporting the conclusions of this article will be made available by the authors, without undue reservation.
