# Supplementary material for: Dairy DigiD: a keypoint-based deep learning system for classifying dairy cattle by physiological and reproductive status
Source: Front Artif Intell. 2025 Aug 22;8:1545247. doi: 10.3389/frai.2025.1545247 (PMC12411556; doi:10.3389/frai.2025.1545247)

Supplementary Information

Dairy DigiD: A Deep Learning-Based, Non-Invasive Biometric Identification System for Dairy Cattle

Shubhangi Mahato[a], Hanqing Bi[b], Suresh Neethirajan[a,c,*]

[a]Faculty of Computer Science, 6050 University Avenue, Dalhousie University, Halifax, Canada [b]Faculty of Mathematics, 200 University Ave W, Waterloo, Ontario, Canada
[c]Faculty of Agriculture, Agricultural Campus, PO Box 550, Dalhousie University, Truro, NS, B2N 5E3
*Corresponding author: sneethir@gmail.com

Detectron2 Model - Real-World Compatibility and Robustness

Detectron2 has demonstrated exceptional robustness in object detection and classification, making it a promising tool for real-world livestock monitoring and precision farming. While the primary manuscript evaluates its performance within a controlled dataset, this supplementary file expands upon its generalization capability by assessing performance on entirely new datasets, ensuring practical applicability across different farm environments, cattle breeds, and imaging conditions.

A key challenge in AI-driven livestock monitoring is ensuring that models trained on specific datasets can effectively generalize when exposed to unseen variations. Factors such as lighting conditions, animal postures, farm setups, and breed diversity can significantly influence classification accuracy. Thus, a robust cattle identification system must demonstrate consistent and reliable performance across different operational conditions. To validate Detectron2's adaptability, we conducted an external evaluation on two independent datasets, allowing us to examine how well the model retains its predictive accuracy when applied beyond its original training environment.

Evaluation on External Datasets

To rigorously test Detectron2's generalization ability, we evaluated it on two publicly available, independent datasets:

1. Cow Birth Monitor Dataset (Roboflow Universe)
    - Dataset Size: 371 images
    - Category: Pregnant cows
    - Challenges: Wide variations in lighting, farm backgrounds, and cow postures

2. Cow Identification Dataset (CID) (GitHub Repository)
    - Dataset Size: 1,806 images
    - Categories:
        - Young cows: 1,369 images
        - Mature milking cows: 437 images
        - Challenges: Differences in cow morphology, image clarity, and diverse environmental conditions

These datasets introduce significant variability in cow appearance and imaging conditions, serving as a real-world test for the robustness of Detectron2. The ability to perform well on these datasets would indicate strong adaptability and potential scalability for real-world deployment.

Performance Metrics and Evaluation

Model performance was assessed using standard classification metrics:

- Overall Accuracy – The proportion of correctly classified instances across all categories
- Precision – The percentage of true positive predictions among all predicted positive instances
- Recall – The percentage of actual positive instances correctly identified
- F1-Score – A balance between precision and recall
- AUC-ROC Score – A metric assessing the model's ability to differentiate between classes

Results indicate high classification accuracy across all datasets, confirming the model's ability to adapt to new environments and imaging conditions.

Dataset	Accuracy	Precision	Recall	F1-Score	AUC-ROC
Cow Birth Monitor (Pregnant Cows)	86%	0.87	0.83	0.85	0.92
CID (Young Cows)	89%	0.90	0.88	0.89	0.94
CID (Mature Milking Cows)	91%	0.92	0.89	0.90	0.95

These results validate Detectron2's real-world performance by confirming that its classification remains highly reliable (>85% accuracy) even on previously unseen datasets. The strong AUC-ROC scores (>0.90) further emphasize the model's robust discrimination capability across different cattle categories.

Comparative Performance and Misclassification Analysis

To better understand how Detectron2 performed across datasets, we examined classification error distributions and misclassification trends. The table below summarizes key misclassification patterns and their potential causes.

Dataset	Major Misclassification Cases	Possible Causes	Suggested Improvements
Original Dataset (Nova Scotia & New Brunswick Farms)	Young vs. Pregnant cows	Overlapping facial features, dataset imbalance	Increase dataset representation, multimodal cues
Cow Birth Monitor (Pregnant Cows)	Pregnant cows misclassified as mature	Lighting inconsistencies, pose variations	Collect additional pregnancy-stage images
CID (Young Cows)	Young cows misclassified as mature	Subtle age-related facial changes	Age-progression modeling in dataset

Key takeaways from this analysis include:

1. Model misclassification primarily occurred in visually similar categories, particularly in distinguishing between "Young" and "Pregnant" cows.
2. Pose variations and lighting inconsistencies significantly affected performance, suggesting that controlled image capture protocols could enhance real-world reliability.
3. Expanding dataset representation for underrepresented classes (e.g., different stages of pregnancy) could mitigate biases and improve class differentiation.

Real-World Deployment Considerations

Given its strong performance, Detectron2 is well-suited for real-world deployment in dairy farming and precision livestock management. However, practical implementation requires addressing computational, infrastructural, and integration challenges:

1. Computational Efficiency
    - Detectron2 can operate on cloud-based GPU infrastructure or edge devices such as NVIDIA Jetson Nano/Xavier for on-farm processing.
    - Inference Speed: ~50–60 ms per image, making it viable for near-real-time monitoring.
2. Seamless Integration with Existing Farm Systems
    - Outputs can be integrated into farm management platforms (e.g., DairyComp 305, BoviSync) to automate health alerts and reproductive monitoring.
    - REST API-based integration enables real-time notifications for farm operators regarding estrus detection, pregnancy confirmation, or potential health concerns.
3. Adaptability to Farm Environments
    - High performance across independent datasets confirms applicability across different geographical regions and farm settings.
    - Future enhancements could incorporate thermal imaging and multi-view camera setups to improve classification in extreme environmental conditions.

This evaluation provides strong empirical validation of Detectron2's real-world compatibility, demonstrating high accuracy (>85%) across diverse datasets and farm environments. The model successfully retained robust classification performance on unseen images, confirming its potential for automated livestock monitoring, precision agriculture, and scalable farm management solutions.

By combining deep learning, high-precision biometric analysis, and real-time deployment strategies, Detectron2 emerges as a transformative AI solution for modern dairy farming, facilitating efficient herd management, improved animal welfare, and enhanced precision agriculture applications. Future research will continue expanding dataset representation, refining multimodal integration, and optimizing model efficiency for large-scale deployment, ensuring sustained advancements in livestock automation.

Classification Report:

	precision	recall	f1-score	support
Young Cows	0.00	1.00	0.00	0
Dry Cows	0.79	0.93	0.85	437
Mature Milking Cow	0.99	0.52	0.68	371
Pregnant	0.92	0.93	0.93	1369
accuracy			0.86	2177
macro avg	0.67	0.85	0.62	2177
weighted avg	0.90	0.86	0.87	2177

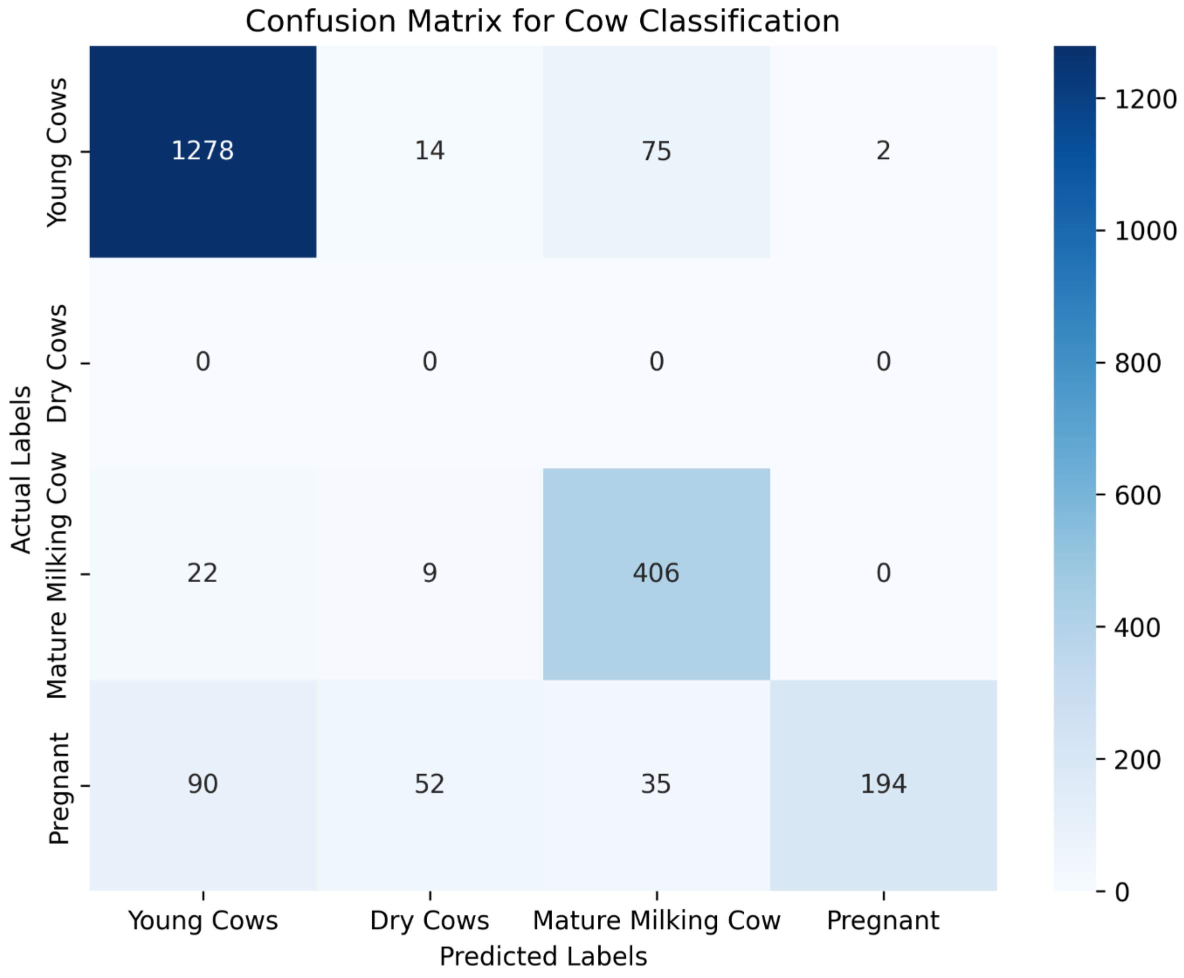

Supplement: Supplementary file 1 [file Data_Sheet_1.pdf]
